# Human–Mosquito Contact: A Missing Link in Our Understanding of Mosquito-Borne Disease Transmission Dynamics

**DOI:** 10.1093/aesa/saab011

**Published:** 2021-05-10

**Authors:** Panpim Thongsripong, James M Hyman, Durrell D Kapan, Shannon N Bennett

**Affiliations:** 1 Department of Microbiology, California Academy of Sciences, 55 Music Concourse Drive, San Francisco, CA 94118, USA; 2 Department of Mathematics, Tulane University, 6823 St. Charles Avenue, New Orleans, LA 70118, USA; 3 Department of Entomology and Center for Comparative Genomics, Institute of Biodiversity Sciences and Sustainability, California Academy of Sciences, 55 Music Concourse Drive, San Francisco, CA 94118, USA; 4 Center for Conservation and Research Training, Pacific Biosciences Research Center, University of Hawai’i at Manoa, 3050 Maile Way, Honolulu, HI 96822

**Keywords:** human–mosquito contact, mosquito biting rate, bite-exposure rate, blood-feeding rate, vector-borne diseases

## Abstract

Despite the critical role that contact between hosts and vectors, through vector bites, plays in driving vector-borne disease (VBD) transmission, transmission risk is primarily studied through the lens of vector density and overlooks host–vector contact dynamics. This review article synthesizes current knowledge of host–vector contact with an emphasis on mosquito bites. It provides a framework including biological and mathematical definitions of host–mosquito contact rate, blood-feeding rate, and per capita biting rates. We describe how contact rates vary and how this variation is influenced by mosquito and vertebrate factors. Our framework challenges a classic assumption that mosquitoes bite at a fixed rate determined by the duration of their gonotrophic cycle. We explore alternative ecological assumptions based on the functional response, blood index, forage ratio, and ideal free distribution within a mechanistic host–vector contact model. We highlight that host–vector contact is a critical parameter that integrates many factors driving disease transmission. A renewed focus on contact dynamics between hosts and vectors will contribute new insights into the mechanisms behind VBD spread and emergence that are sorely lacking. Given the framework for including contact rates as an explicit component of mathematical models of VBD, as well as different methods to study contact rates empirically to move the field forward, researchers should explicitly test contact rate models with empirical studies. Such integrative studies promise to enhance understanding of extrinsic and intrinsic factors affecting host–vector contact rates and thus are critical to understand both the mechanisms driving VBD emergence and guiding their prevention and control.

The emergence and intensification of novel and established vector-borne diseases (VBDs) are an increasing threat to global public health (World Health Organization 2014, 2017). Eighty percent of the world population is currently at risk of contracting VBDs, and over 700,000 people die from them annually (World Health Organization 2017). Despite significant progress in controlling malaria, the disease still causes >400,000 deaths annually (World Health Organization 2019). Dengue, the most prevalent mosquito-borne viral disease, threatens hundreds of millions of people yearly despite significant control efforts (Bhatt et al. 2014, Stanaway et al. 2016). Significant outbreaks of other mosquito-borne diseases, including chikungunya, West Nile, and Zika, add to the growing public health burden (Weaver and Reisen 2010, Mayer et al. 2017). Infections transmitted by other biting insects, including blackflies (e.g., onchocerciasis), sandflies (e.g., leishmaniasis), triatomine bugs (e.g., Chagas disease), tsetse flies (e.g., trypanosomiasis), and ticks (e.g., Lyme disease), cause over 26 million cases annually (World Health Organization 2017).

For vector-borne pathogens, vector bites are critical for transmission, and host–vector contact rate is consistently the most important parameter determining disease risk according to studies using mathematical models (e.g., Chitnis et al. 2008, Ellis et al. 2011, Gao et al. 2016). Yet despite the key role that contact rates have in driving disease transmission, most research focuses *primarily* on vector density rather than contact rate in assessing VBD dynamics (e.g., Martens et al. 1995, Sanchez et al. 2006, Beck-Johnson et al. 2013, Sang et al. 2014, Kraemer et al. 2015, Leta et al. 2018, Romeo-Aznar et al. 2018).

Vector density, or the number of vectors per unit area, is an essential VBD transmission factor, but cannot solely predict the emergence and spread of VBDs. Consideration of the vector component of disease transmission alone cannot generate a complete picture of how changes in host behavior, vector feeding habits, the environment, and societal factors influence disease transmission.

For example, warming global temperatures, on the one hand, could increase transmission due to the expansion of mosquito habitat and distribution in some locations (Alto and Juliano 2001, Fischer et al. 2014, Kamal et al. 2018, Mordecai et al. 2019, Ryan et al. 2019). On the other hand, warming temperatures could reduce transmission if people spend more time sheltering indoors where there are fewer mosquitoes (e.g., in air-conditioned buildings). In an area where open housing is more common, they would be consistently exposed to mosquito bites, leading to increased disease risk with mosquito expansion. Thus, even if global warming increases vector density in a region, transmission risk will depend upon the living conditions and vector access to humans (Fig. 1). Even though studies show that vector distribution and number varies significantly across land-use types, such as rural versus urban (Thongsripong et al. 2013, Ndenga et al. 2017) or socioeconomic factors (Reiter et al. 2003, Feria-Arroyo et al. 2020), none exist to show how such differences directly impact measured human–vector contact rate.

**Fig. 1.**
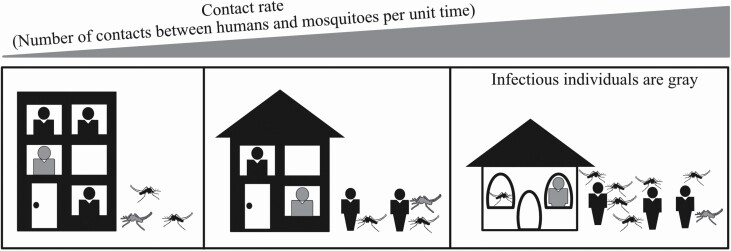
Schematic diagram showing the potential impact of human behavior/distribution, socioeconomic factors, and mosquito density on contact rate and mosquito-borne disease transmission. The infectious mosquitoes and individuals are shown in gray. At the lowest extreme for contact rate (left panel), air conditioning availability creates a significant barrier between outdoor mosquitoes and humans opting for indoor environments. At the highest extreme (right panel), open-air housing, combined with higher human outdoor activity and increased mosquito density, leads to high contact rates indoors and outdoors.

As half the world’s population repeatedly experiences multiple outbreaks of VBDs, we continue to have a limited understanding of VBD transmission dynamics and the importance of host–vector contact as a significant factor in disease transmission. This overlooked gap impedes our comprehensive understanding of how diseases emerge and spread, limiting our ability to control them. We propose a new modeling framework that will also help direct empirical studies on contact rate, of which there are few (Scott et al. 2000, Monroe et al. 2019, Thongsripong et al. 2020). This framework focuses on host–vector contact to broaden our insights into how contact dynamics impact mosquito-borne disease transmission dynamics and are influenced by host and vector biology, and socioecological factors.

This article synthesizes current knowledge on host–vector contact focusing on how contact dynamics drive VBD transmission and subsequent risk. The questions driving the article include how do host-vector contact dynamics drive VBD transmission and subsequent VBD risk? What are the factors that drive host-vector contact dynamics? And how can we capture and explain these dynamics in the field? To answer these questions, we first establish a framework for how humans and mosquitoes interact to exchange pathogens via mosquito bites (‘What is Human–Mosquito Contact?’ section). This includes providing clear biological and mathematical definitions for bite-related terms. Next, we review the literature on the intrinsic biological and extrinsic environmental factors that can influence human–mosquito contact rates, where our modeling framework will provide the most insights (‘Factors Influencing Host–Vector Contact’ section). Finally, we characterize how human–mosquito contact determines disease risk by incorporating contact rate into mechanistic transmission models (‘Mathematical Models of Host–Vector Contact Rates’ section). In doing so, we describe how models can capture changes in host–vector contact rates as a function of vector and host populations and their behaviors. Indeed, by elucidating mathematical frameworks to account for contact dynamics, we identify the critical gaps in biological data that could otherwise be used to test these models. This process can help us to pinpoint the most important factors influence contact dynamics in VBD. We also identify the need to bridge biological data with mathematical models. Our framework for incorporating contact rates provides insights gained to understand transmission dynamics and disease control and demonstrates that host–vector contact is the key to understanding mechanisms of disease spread.

## What Is Human–Mosquito Contact?

### Biological Definition of Human–Mosquito Contact

**Fig. 2.**
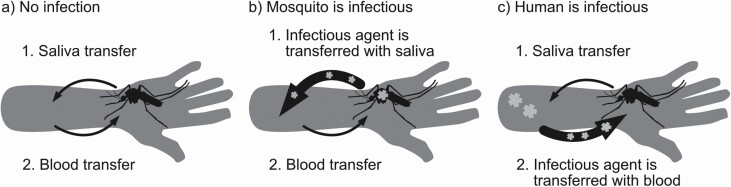
Two processes occur sequentially during contact between a human and a mosquito: 1) biting and release of saliva and 2) blood-sucking (a). The two processes represent two separate and distinct directions for pathogen transmission. When the mosquito is infectious, the pathogen is transferred to the human with its saliva (b). When the human is infectious, the pathogen is transferred to the mosquito with human blood (c).

Biting and blood-feeding are two distinct processes that occur *sequentially* during human–mosquito contact (Fig. 2a; Walker and Edman 1985a, Choumet et al. 2012). During the biting (or probing) process, a mosquito searches for blood vessels by using the fascicle to penetrate the host skin. While biting, it releases saliva containing immunomodulators and anticoagulants (Ribeiro 1987, Choumet et al. 2012). The mosquito might have to probe several times on a single host before successfully lacerating a vessel (Griffiths and Gordon 1952, Walker and Edman 1985a). The blood-feeding process follows once a blood vessel is found. Blood is then sucked up through the mosquito’s stylet (Friend and Smith 1977, Choumet et al. 2012). Because blood-feeding is not the same as biting, the mosquito’s blood-feeding rate (the number of blood meals per mosquito per unit time) can differ from its biting rate (the number of bites, with or without a bloodmeal, per mosquito per unit time).

It is pertinent to differentiate between blood-feeding and biting because two directions of pathogen transmission are possible during a contact. In the first direction (Fig. 2b), when an infectious mosquito bites a susceptible person, it can transmit the pathogen in its saliva to the human during probing (Chamberlain and Sudia 1961). Blood-feeding, a process that happens after saliva is released, is not required for successful pathogen transmission from mosquito to human. Matsuoka et al. (2002) found that mice can develop malaria when infectious *Anopheles stephensi* (Liston; Diptera: Culicidae) probed into the mouse skin but were not permitted to take blood. We are not aware of any experiments to confirm if this same phenomenon happens in other pathogens.

In the other direction, a mosquito becomes infected after probing *and* imbibing blood from an infectious person (Fig. 2c). The blood containing the pathogen must be transported into the mosquito midgut before the pathogen can replicate (Carrington and Simmons 2014).

### Mathematical Definition of Human–Mosquito Contact

For mosquito species *v* and vertebrate host species *h*, we define vector–host contact rate (***c***_***_vh_***_) as the total number of contact events between individuals of species *v* and species *h* per unit time for a given area of interest. These contact events include either blood-feeding or biting, which could allow for pathogen transmission. This contact rate is distinct from the mosquito’s blood-feeding rate (***b***_***_vh_***_), which we define as the average number of blood meals a *single mosquito* attains from hosts per unit time for a given area of interest. Similarly, contact rate is distinct from bite-exposure rate (***e***_***_vh_***_), which we define as the average number of mosquito bites (with or without blood-feeding) that a *single host* experiences per unit time for a given area of interest (Thongsripong et al. 2020). We define the term *biting rate* as the average number of bites (with or without blood-feeding) *a single mosquito* takes from hosts per unit time for a given area of interest. Thus, it is distinct from both blood-feeding and bite-exposure rates. The blood-feeding rate, biting rate, and bite-exposure rate are per capita rates, whereas the contact rate is not. Although these terms are biologically distinct, they have often been used interchangeably in the literature, leading to ambiguity.

The blood-feeding and bite-exposure rates play complementary roles in VBD transmission. In ‘Mathematical Models of Host–Vector Contact Rates’ section, we describe how the blood-feeding rate determines the rate that the pathogen is transmitted from the host to the mosquito (via blood transfer). The bite-exposure rate determines the rate that the pathogen is transmitted from mosquito to host (via saliva transfer). Although these rates are biologically distinct, it can be impractical to model them separately since field data are seldom available to differentiate between bite-exposure and blood-feeding rates.

## Factors Influencing Host–Vector Contact

**Fig. 3.**
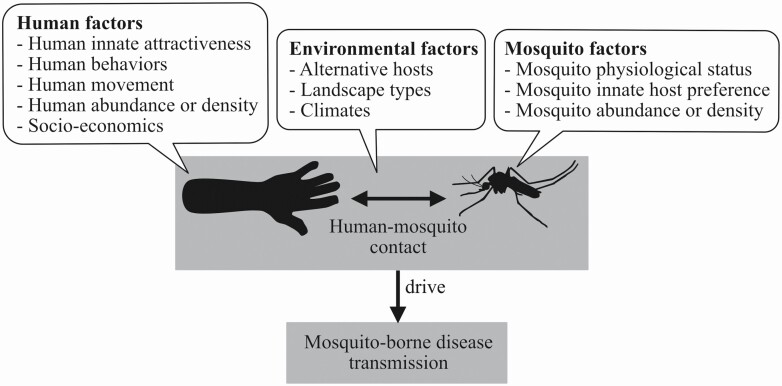
Human–mosquito contact is a central driver of disease transmission, while mosquito density is only a factor determining disease risk.

The general propensity to focus on mosquito density as the primary driver of disease risk overlooks the biological contributions of vector, host, pathogen, and environmental interactions to mosquito-borne disease transmission dynamics. Mosquito-borne pathogens are transmitted within a complex socio-environmental system. The system consists of multiple but integrated and interactive components, including hosts, vectors, pathogens, and other socio-environmental factors (exemplified in Fig. 3). The nonlinear interactions among these factors influence host–vector contact and drive system-level dynamics of disease transmission. Thus, host–vector contacts are the *key* events where the main actors interact physically. These rates are an ideal starting point to begin teasing apart the complex interplay among variables.

### Mosquito Gonotrophic Cycle Length

When female mosquitoes emerge, there exists a short maturation period during which they do not blood-feed; instead, they prefer sugar-rich meals (Klowden 1990, Omondi et al. 2019). This lasts from 1 to 3 d on average (Scott and Takken 2012). Eventually, after blood-feeding, they find a suitable place to rest in the days that follow to digest blood and develop a clutch of eggs (Klowden and Briegel 1994, Duvall 2019). Host-seeking behaviors can become suppressed during this period (Duvall 2019). Once eggs are laid, they often start to host-seek again. This simplified cycle of blood-feeding and egg-laying, known as the gonotrophic cycle, repeats throughout the female mosquito’s lifetime.

The length of the gonotrophic cycle is often used to determine the rate of mosquito blood-feeding. On average, the mosquito’s gonotrophic cycle lasts approximately 2–4 d (Pant and Yasuno 1973, Delatte et al. 2009, Duvall 2019). If a female mosquito takes one bloodmeal at every cycle without any delays, her blood-feeding rate is then the reciprocal of the gonotrophic cycle duration. For example, if the average gonotrophic period lasts 2 d, then the mosquito per capita blood-feeding rate would be 0.5 blood meals per day (i.e., one bloodmeal every two days). This is among the most common ways that the blood-feeding rate (and biting rate) is derived and incorporated in deterministic disease transmission models (Esteva and Vargas 1998, Smith et al. 2012).

### Climatic Factors

In reality, environmental variables can alter the length of the gonotrophic cycle, impacting mosquito biting and blood-feeding rates (Gillies 1953, Klowden and Lea 1978, Foster 1995, Afrane et al. 2005, Gu et al. 2006, Delatte et al. 2009, Gu et al. 2011). Laboratory studies have demonstrated that warming temperature, to a certain degree, shortens the gonotrophic cycle for *Anopheles* and *Aedes* (Rúa et al. 2005, Lardeux et al. 2008, Delatte et al. 2009, Carrington et al. 2013, Paaijmans et al. 2013, Eisen et al. 2014, Goindin et al. 2015). However, the shape of the relationship between temperature and length of the gonotrophic cycle is poorly defined, and studies confirming this in the field are rare. One semifield study comparing mean indoor temperature between forested and deforested areas in Western Kenya showed an increased mean indoor temperature in the deforested area shortened *Anopheles gambiae’s* (Giles; Diptera: Culicidae) gonotrophic cycles by up to 1.7 d (Afrane et al. 2005). However, it has not been determined if a shorter gonotrophic cycle increases a mosquito’s blood-feeding and biting rates.

Cold temperature can also directly reduce mosquitoes’ biting rates because it reduces their host-seeking and flight activity (Rowley and Graham 1968). Similarly, windy weather can inhibit host-seeking flight or affect the olfactory cues from vertebrate hosts, thus impacting biting rates (Service 1980, Gibson and Torr 1999, Hoffmann and Miller 2002).

Rainfall can theoretically impact mosquitoes’ blood-feeding rates by altering the number of available oviposition sites. During a dry period, when oviposition sites were limited, females retained their eggs until they found an acceptable site (Smartt et al. 2010, Brown et al. 2014). Egg retention was associated with suppressing host-seeking behavior and blood-feeding (Klowden and Lea 1979a, Klowden 1990, Bowen 1991, Johnson and Fonseca 2014). In a time series analysis of field-collected *Culex nigripalpus*, the number of gravid females was associated negatively with daily rainfall (Day et al. 1990). The authors suggested that intermittent rainfall could have delayed oviposition, increased longevity, and synchronized the blood-feeding. These events could have combined to enhance the transmission of St. Louis encephalitis. After a colony of *An. gambiae* experienced a weeklong oviposition-site deprivation in a laboratory study, they exhibited a reduced feeding rate relative to the control group (Artis et al. 2014). Field-based studies confirming the relationship between rainfall and mosquito blood-feeding rate are needed.

Humidity can also influence the blood-feeding rate. A recent study investigating the effect of dehydration on mosquito blood-feeding activity found that the propensity of *Culex pipiens* (Linnaeus; Diptera: Culicidae), *Aedes aegypti* and *Anopheles quadrimaculatus* (Say; Diptera: Culicidae) to blood-feed increased significantly after mosquitoes were kept in a low-humidity environment and had lost at least 10% of water content (Hagan et al. 2018).

### Vector Multiple Blood-Feeding Behaviors

The simplified way of deriving blood-feeding and biting rates from gonotrophic cycle length does not fit within the more complicated cycle of some vector species. In contrast to the common assumption of one bloodmeal per cycle, *Anopheles*, *Aedes*, and *Culex* can take multiple blood meals within a single gonotrophic cycle, a behavior known as multiple blood-feeding. For example, small female *An. gambiae* lack enough metabolic reserves for ovarian development. They require at least two blood meals to complete their first gonotrophic cycle (Scott and Takken 2012). Other *Anopheles* species such as *Anopheles funestus* (Giles; Diptera: Culicidae), *Anopheles albimanus* (Wiedemann; Diptera: Culicidae), *Anopheles arabiensis* (Patton; Diptera: Culicidae), and *Anopheles freeborni* (Aitken; Diptera: Culicidae) have also been observed to ingest multiple blood meals in a cycle (Klowden and Briegel 1994, Norris et al. 2010, Scott and Takken 2012). Similarly, *Ae. aegypti*, *Aedes albopictus* (Skuse; Diptera: Culicidae), and many *Culex* species exhibit varying degrees of multiple blood-feeding (Scott et al. 1993, Anderson and Brust 1995, Arunachalam et al. 2005, Muturi et al. 2008, Delatte et al. 2010). Blood-fed and gravid female *Ae. aegypti* and *Ae. albopictus* continue to seek hosts; thus, their host-seeking behavior is not suppressed (Scott and Takken 2012, Kim et al. 2017). In one experiment, blood-fed *Cx. tarsalis* (Coquillett; Diptera: Culicidae), *Cx. restuans* (Theobald; Diptera: Culicidae), and *Cx. nigripalpus* (Theobald; Diptera: Culicidae) were collected after spending overnight in box traps baited with a pair of quail (Anderson and Brust 1995). Approximately 5–30% of the mosquitoes ingested blood from both quail.

The observations of multiple blood-feeding in the field contrast with many early observations in the laboratory where *Ae. aegypti* and *Ae. albopictus* refrain from taking another bloodmeal after a full one is ingested (Klowden and Briegel 1994). Early works with *Ae. aegypti* established two distinct physiological mechanisms inhibiting further feeding once females ingested blood. The first mechanism, *distention-induced inhibition,* triggered by abdominal stretch receptors, becomes activated upon blood-feeding (Klowden and Lea 1978, 1979a). Once blood is digested and the eggs develop, the second mechanism, *oocyte-induced inhibition,* becomes activated and continues to suppress host-seeking via humoral processes (Klowden and Lea 1979c, Klowden 1981).

The assumption of one bloodmeal per cycle mirrors how mosquitoes are reared in the insectary and could be artifactual (Klowden 1994). Typically, mosquito colonies are given blood to repletion via anesthetized hosts or artificial feeders and then allowed to oviposit before given another round of blood. In the field, the hosts are alive and active; feeding to repletion is not always possible. Klowden and Lea (1978) showed that abdominal distention inhibited *Ae. aegypti*’s host-seeking behavior only when blood volume was above a certain threshold. The mosquitoes that ingested partial meals (≤3 μl in *Ae. aegypti;*Klowden 1994) continued to host-seek. In addition, not all mosquitoes that ingest a small meal will develop eggs (Xue et al. 1995), and the oocyte-induced inhibition would never take place. Even when the eggs develop, smaller meals can be digested rapidly. Thus, there can be a brief period where the distention-induced inhibition is lifted before oocyte-induced inhibition is initiated (Klowden 1994).

Other vectors’ endogenous factors such as age (Klowden and Lea 1984), nutrition, parity status, and male diet can influence the multiple feeding behavior (Klowden 1994). For example, Farjana and Tuno (2013) showed that multiple feeding behavior of *Ae. albopictus* was negatively correlated with body size. Malnourished gravid *Ae. aegypti* show less inhibition to blood-feed (Klowden 1986). Even though blood and sugar feeding are antagonistic, they are interchangeable as energy sources (Foster 1995). In fact, many *Aedes* and *Anopheles* prefer blood to sugar when blood sources are available (Edman et al. 1992, Scott and Takken 2012).

Thus, the assumption of one bloodmeal per gonotrophic cycle might not hold in natural conditions. Important factors, such as host defensive behaviors and mosquito physiological status, play essential roles in determining mosquito biting and blood-feeding rates. Using gonotrophic cycle length alone cannot result in an accurate estimate of the human–mosquito contact rate.

### Mosquito Innate Host Preference

Infection permissiveness and infectiousness vary across individual mosquitoes and species. Therefore, a nonrandom host–vector contact pattern significantly influences disease transmission. For example, contacts might concentrate on a highly infectious host, creating a superspreading event (Woolhouse et al. 1997, Cooper et al. 2019). The variation in contact patterns in mosquito-borne diseases could be due to host availability and movement (Adams and Kapan 2009, Stoddard et al. 2009, Seyoum et al. 2012, Thiemann and Reisen 2012, Moiroux et al. 2014, Acevedo et al. 2015, Finda et al. 2019), host defensive behavior (‘Host Defensive Behaviors’ section), and innate vector preference for certain hosts.

Choice experiments demonstrate the innate preference of many *Anopheles, Aedes*, and *Culex* vectors to bite certain host species or individuals (Costantini et al. 1998, Dekker and Takken 1998, Mboera and Takken 1999, Duchemin et al. 2001, Pates et al. 2001, Simpson et al. 2009, Delatte et al. 2010). In these experiments, selective mosquito behavior was less likely to be influenced by host density, availability, or defensive behavior. Therefore, mosquitoes target-specific hosts based on attributes such as odor, heat, body surface, and other physiological and genetic characters (Takken and Verhulst 2013).

Infection status is another host attribute that mosquitoes can use, and this significantly impacts transmission dynamics. Cornet et al. (2013b) showed that when mosquitoes were provided a choice between birds acutely infected, chronically infected, and uninfected, with *Plasmodium relictum* (all birds were immobilized), the chronically infected birds attracted significantly more vectors. Anophelines also showed enhanced attraction to mice infected with rodent malaria *P. chabaudii* (De Moraes et al. 2014). This attraction appeared to be mediated by an overall elevation of volatiles emitted from infected mice compared to uninfected (De Moraes et al. 2014). Human malaria (*P. falciparum* and *P. vivax*) also rendered humans more attractive to *An. gambiae* (Koella et al. 1998, Lacroix et al. 2005) and *An. darlingi* (Batista et al. 2014). A follow-up study showed that *P. falciparum* produced a chemical compound that triggered human red blood cells to release more CO_2_, aldehydes, and monoterpenes, which collectively enhanced vector attraction and stimulated vector feeding (Emami et al. 2017). The effect of hosts’ viral infection status on mosquito biting preference is currently unknown.

Vector infection status can also influence behavior and human–vector contact rate. *Anopheles* infected with *Plasmodium* show species-specific changes in olfactory sensitivity that impacts biting behavior (Stanczyk et al. 2019). Dengue virus infection in vectors can alter their feeding behavior and frequency. *Aedes aegypti* infected with dengue virus serotype 3 spent longer probing and acquiring blood meals (Platt et al. 1997). The authors suggested that the longer feeding was likely to be interrupted, increasing the chance that an infected mosquito would probe on additional hosts. However, other studies found no evidence that the dengue virus serotype 2 impaired the blood-feeding efficiency of *Ae. aegypti* (Putnam and Scott 1995, Sim et al. 2012). Cornet et al. (2013a) found that both infected and uninfected mosquitoes were attracted toward malaria-infected birds. Schaub (2006) found parasitic alterations of other arthropods’ biting behaviors affected disease transmission in plaque, tick-borne encephalitis, trypanosomiasis, and Leishmaniasis.

### Host Defensive Behaviors

Contact between host and vector is a two-way process. For successful contact, the vector needs to initiate the bite, and the host needs to allow the bite to occur. The vector’s propensity to bite alone does not determine the contact rate. Host availability and defensiveness, among other factors, determine whether contact occurs to allow both transmissions from an infected mosquito to the human (salivary transfer of infectious agent) and from an infected human to the mosquito (blood transfer of infectious agent).

Walker and Edman (1985b) recorded in the laboratory that when *Ae. triseriatus* (Say; Diptera: Culicidae) failed to take a bloodmeal from a human hand due to multiple interruptions, they eventually ceased attacking. The sucrose-starved mosquitoes were significantly less persistent, and they suggested that carbohydrate reserve was necessary for biting persistence. Other studies illustrated the similar relationships between the attack rate or biting persistence with the energy state and body size of *An. gambiae* (Roitberg et al. 2010, Reid et al. 2014) and *Ae. aegypti* (Nasci 1991).

In another laboratory study, when *Cx. nigripalpas* density was gradually increased, there was a decrease in the overall engorgement rate, but a corresponding increase in the proportion of blood taken from a tolerant host (Edman et al. 1974). The authors argued that the defensive activities of birds and small mammals, rather than any lack of attractiveness, were responsible for lower mosquito engorgement rates. Another laboratory study observed that when *Ae. triseriatus* density increased, host defensive behaviors increased, resulting in a decrease in mosquito feeding success (Walker and Edman 1986). Rabbit grooming interfered with mosquito blood-feeding and reduced the number of successful feeds of many *Aedes* species, including *Ae. aegypti* (Klowden and Lea 1979b, Waage and Nondo 1982). Day and Edman (1984) recorded the mortality and engorgement rate of five mosquito species after they were released into cages holding different restrained and unrestrained host species. Unrestrained young chickens and rodents were able to capture and eat some mosquito species better than others. A colony strain of *Cx. nigripalpas* suffered higher mortality compared with a wild colony.

Lyimo et al. (2012) evaluated mosquitoes’ feeding success in a semifield environment. They found that *An. arabiensis* had a greater feeding success when applied directly to host skin than when foraging on unrestricted hosts in five out of six host species. On the other hand, *An. gambiae* s.s. obtained blood from both free and restrained hosts with similar success from four out of six host species. It is possible that cattle, horses, and other large mammals are less defensive than small mammals and birds (Lyimo et al. 2012). Sota et al. (1991) showed that even though a pig’s defensive behavior reduced the *Cx. tritaeniorhynchus* (Giles; Diptera: Culicidae) attack rate, the effect was only temporary, and the overall number of mosquitoes per pig was not significantly affected by the pig’s activity. Another study found that avian defensive behavior increased proportionately with mosquito density; however, it decreased after the birds were exposed to mosquitoes multiple times (Darbro and Harrington 2007). Blood-feeding success was negatively correlated with chicks’ defensive behavior, but this was not the case for house sparrows. There was a higher feeding success rate on house sparrows and a higher percentage of partial bloodmeal on chicks, and birds of both species were observed to eat around 9% of the mosquitoes. The blood-feeding-related mortality rate of mosquitoes in nature is unknown.

Direct field observations of animal defensive behavior against mosquito blood-feeding are rare. In a semifield environment, Hodgson et al. (2001) presented *Culiseta melanura* (Coquillett; Diptera: Culicidae) mosquitoes with two choices of caged birds to feed on each night. They observed that, when given a choice, the mosquitoes fed meagerly on starlings and abundantly on robins. The birds were free to move within their cages, and the authors suggested that the starling’s defensive behaviors interrupted the blood-feeding.

Humans have more options than other animals to protect themselves against insect bites and mosquito-borne diseases. In addition to movement, they might use protective dwellings (Lwetoijera et al. 2013, Tusting et al. 2015, Killeen et al. 2019, Donnelly et al. 2020), bed nets (Lindsay et al. 1989, Clarke et al. 2001, Killeen et al. 2006), clothing (Schoepke et al. 1998, Banks et al. 2014, Londono-Renteria et al. 2015), and mosquito repellents (Brown and Hebert 1997, Patel et al. 2016, Islam et al. 2017, Maia et al. 2018). However, a direct relationship between bite protection behaviors and human–mosquito contact rates has rarely been investigated (Moiroux et al. 2014, Monroe et al. 2019, Thongsripong et al. 2020). Moreover, climatic and socioeconomic factors can influence human protective behavior against mosquito bites, leading to variation across settings (Charlwood et al. 1995, Hill et al. 2013, Gryseels et al. 2015, Aberese-Ako et al. 2019).

Among the most effective and widely utilized tools to protect against mosquito bites, especially night biters, are bed nets. Insecticide-treated nets (ITNs) and Long-lasting Insecticide-treated nets (LLINs) are standard components of many malaria control programs (Hemingway 2014, Benelli and Beier 2017). ITNs and LLINs can reduce indoor density, indoor biting, survival, and infectiousness of mosquito populations (Lindsay et al. 1989, Mathenge et al. 2001, Takken 2002, Gimnig et al. 2003, Lim et al. 2011, Mutuku et al. 2011), leading to a reduction in malaria incidence and improved public health outcomes (Choi et al. 1995, Lengeler 2004, Lim et al. 2011, Yang et al. 2018). However, multiple factors such as climate, socioeconomic status, and the perception of low mosquito density can reduce bed net usage, impacting its efficacy (Atieli et al. 2011, Pulford et al. 2011). Studies occasionally showed that Anophelines change their feeding behaviors (shifts in feeding time, feeding site, and blood hosts) after the nets were employed (Russell et al. 2011, Moiroux et al. 2012, Sherrard-Smith et al. 2019). This could result in an overall higher bite-exposure rate and malaria transmission rate in some cases (Thomsen et al. 2017).

Studies confirming a direct impact of mosquito bite protection on the overall contact rate are rare (Mitchell et al. 2020, Monroe et al. 2020). Such studies are often in laboratory settings, for example, investigations on the efficacy of repellents and insecticide-treated clothing in reducing human-vector contact (Fradin et al. 2002, Ogoma et al. 2012, Lupi et al. 2013). Some field studies have indirectly measured contact rates in natural settings (Maia and Moore 2011, Van Roey et al. 2014, Londono-Renteria 2015), but few have explicitly measured how protective measures directly impact human–vector contact rate in the field (Lillie et al. 1988, Vaughn and Meshnick 2011, Rossbach et al. 2014).

Likewise, few studies investigate how humans actively interrupt mosquito bites, which is particularly important for day biters, and those that exist focused on malaria transmission (see review by Monroe et al. 2019). A study by Read and Rooker (1994) employed a unique study design to determine the level of mosquito density that research participants considered annoying and to characterize their behavioral responses. They compared the perceived bite number and observed defensive behavior of each research participant during a 5-min blind test period with a concurrent mosquito count from an adjacent human-baited drop-net trap. The frequency of scratching, rubbing, waving or brushing, and slapping or swatting all increased with both trap count and self-reported bite count. The overall rating for annoyance and future repellent use increased with both trap count and reported bite number, whereas the overall rating for anticipated outdoor time decreased. The authors noted that individual response and observed defensive behavior were highly variable at the low trap counts relative to the high trap counts.

Only a few studies explore the reliability of using perceived mosquito bite-exposure to represent actual mosquito activity. We are unaware of any studies that determine the relationship between perceived and actual bite-exposure rates. A survey study based in the southern United States found that self-reported mosquito bite-exposure rates could reflect mosquito biting density in their study sites (Thongsripong et al. 2020). Another study that compared human annoyance with several measures of mosquito abundance in northeast Italy found a close relationship between the two (Carrieri et al. 2012). In contrast, a weak association between entomological activity measured using ovitraps and perceived nuisance has also been reported (Gaillard et al. 2019). The subjectivity of self-reported bite-exposure could introduce bias in the prediction of actual bite exposure. Even so, a person’s perceived bite-exposure and bite tolerance threshold can influence their protective and defensive behaviors and impact their actual bite exposure. In a study by Carrieri et al. (2012), the number of bites considered intolerable by the interviewee was, on average, five bites per day, similar to previous studies in other locations (John et al. 1987, Morris and Clanton 1988).

In conclusion, vector, host, pathogen, and the environment interact to collectively determine the dynamics of host–vector contact rates and patterns that drive disease transmission. Although many mathematical models use a simplifying assumption that vectors possess a constant biting rate regardless of host density or behavior, this might be far from the case in the real world. Highly dynamic host behaviors significantly impact mosquito feeding success and influence the contact rate. Using only mosquito density to determine disease risk could lead to a biased prediction of transmission outcome.

In the next section, we show how human–mosquito contact determines disease transmission when incorporated in a classic mechanistic transmission model. Also, we explore alternative models proposing various ecological mechanisms that influence host–vector contact rate and pattern, impacting disease transmission.

## Mathematical Models of Host–Vector Contact Rates

### Contact Rates in a Classic Epidemiological Compartmental Model: Frequency-Dependent Transmission

Deterministic compartment models describe the dynamic spread of infections among individuals belonging to different compartments represented by a standard notation, such as *S* for ‘Susceptible’, *I* for ‘Infectious’, and *R* for ‘Recovered’. The total population size *N* is the sum of individuals from all compartments. Because this review focuses on contact, we only discuss how individuals of type S convert to type I. This conversion rate equals the rate that the pathogen spreads in the host population and is characterized by the force of infection (λ). The force of infection is the *per capita* rate at which susceptible individuals contract an infection (Choisy et al. 2007). In other words, λ represents the number of new infections per susceptible individual per unit time. Hence, the rate at which the infected individuals are produced per unit time in the population is *λS*, where *S* is the number of susceptible individuals.

In its most basic form, λ is a product of three variables: 1) the *per capita* contact *rate* or the number of contacts an individual has per unit time, 2) the *proportion* of the contacts that are with an infectious host, and 3) the *proportion* of the contacts with an infectious individual that lead to successful transmission (Begon et al. 2002). If every host is equally likely to be bitten, then in a well-mixed population the proportion of the contacts with an infectious host equals the infection prevalence in the population (*I*_*h*_*/N*_*h*_). The proportion of the infectious contacts that lead to infection is derived from experimental or observational studies and is usually assumed to be constant.

In a VBD system, there are at least two host types that the pathogen infects successively: an invertebrate host *v* (we call ‘vector’), and a vertebrate host *h* (we call ‘host’). Thus, there are two forces of infection: one from vector to host (*λ*_*v → h*_) and another from host to vector (*λ*_*h → v*_).

The force of infection from host to vector, *λ*_*h → v*_, is a product of 1) the per capita contact rate that a vector experiences (mosquito’s blood-feeding rate, *b*_*vh*_), 2) the proportion of the contacts that are with an infectious host, which for random mixing equals to *I*_*h*_*/N*_*h*_, and 3) the proportion the vectors that become infected after blood-feeding (*v*_*v*_) on an infected host,


$$\begin{array}{l} \lambda_{h\;\to \;v}=b_{vh}\cdot \frac{I_{h}}{N_{h}}\cdot v_{v\;\;\;}. \end{array}$$ (1)

Note that this formulation assumes that every host is equally likely to be bitten.

The force of infection from vector to host, *λ*_*v →* h_ is a product of 1) the per capita contact rate that a host experiences (bite-exposure rate, with or without blood-feeding, *e*_*vh*_), 2) the proportion of the bites that are from an infectious vector, which for a well-mixed population is *I*_*v*_*/N*_*v*_, and 3) the proportion of these bites that result in infection (*v*_*h*_),


$$\begin{array}{l} \lambda_{v\;\to \;h}=e_{vh}\cdot \frac{I_{v}}{N_{v}}\cdot v_{h} \end{array}$$ (2)

Traditionally in VBD transmission models, the mosquito blood-feeding biting rates for the vectors are assumed to be constant; each mosquito takes the same number of blood meals per unit time regardless of density (Anderson and May 1991). Because the total number of times a mosquito bites a host must equal the total number of times the hosts are bitten by a mosquito, the balance equations require that $N_{v}b_{vh}=N_{h}e_{vh}$. This relationship is used to define the bite-exposure rate as a function of vector–host ratio and their blood-feeding rate, e_*vh*_ = *b*_*vh*_(*N*_*v*_*/N*_*h*_). Thus, one needs to keep in mind that both the blood-feeding and bite-exposure rates cannot be constant unless the vector–host ratio is also constant. Thus, equation (2) can be rewritten as:


$$\begin{array}{l} \lambda_{v\;\to \;\;h}=e_{vh}\cdot \frac{I_{v}}{N_{v}}\cdot v_{h}\;\;=b_{vh}\cdot \frac{N_{v}}{N_{h}}\cdot \frac{I_{v}}{N_{v}}\cdot v_{h}=\;b_{vh}\cdot \frac{I_{v}}{N_{h}}\cdot v_{h} \end{array}$$ (3)

The contact rate components, $\;\;\;b_{vh},and\;e_{vh}$, in each force of infection depends on the size of each population. For example, when there are few hosts and many vectors, then the *per capita* contact rate for the vectors is usually assumed to be almost independent of the population size of the hosts. However, the *per capita* contact rate for the vectors is assumed to increase as the population of hosts increases. The opposite is true when there are many hosts and few vectors.

Each force of infection, λ, contains two components that are related to the human–mosquito contact. The first component of contact is embedded in the per capita contact rate (the bite-exposure rate and the blood-feeding rate). The second component is the proportion of contacts that are with an infectious host or vector. Assuming that the mixing pattern between host and vectors is homogeneous, the proportion of times that the biting vector encounters an infectious host is *I*_*h*_*/N*_*h*_, where *I*_*h*_ and *N*_*h*_ is the number of infectious and total host, respectively. The proportion of times that a host was bitten by an infectious vector can be derived similarly.

While a mosquito’s innate propensity to blood feed or a host’s behavioral response to bites determines the frequency of contacts, the spatial contact structure between the host and vector determines the proportion of contacts with infectious individuals.

### Contact Rates as a Function of Density

While most classic vector–borne disease transmission models assume constant blood-feeding and biting rates, there are dynamic biting models where vector blood-feeding rates vary with host and vector density. These models include a mechanistic model derived from the ecological theory called functional response (Antonovics et al. 1995, Miller et al. 2016, Demers et al. 2018) and a model which derives a contact rate based on a vector’s demand for and hosts supply of contacts (Chitnis et al. 2008, Manore et al. 2015).

#### Functional Response

The term *functional response* describes, in its original usage, the relationship between predation rate (the number of prey consumed per predator per unit time) and prey density (Solomon 1949, Holling 1965). However, its extended application includes consumer types other than predators such as filter feeders (Jeschke et al. 2004), herbivores (Spalinger and Hobbs 1992, Gross et al. 1993), and parasites/parasitoids (Hassell et al. 1977, Antonovics et al. 1995, Fernández-arhex and Corley 2003). Thus, functional response broadly applied in any consumer–resource system explains the influence of resource abundance on the rate of its consumption.

**Fig. 4.**
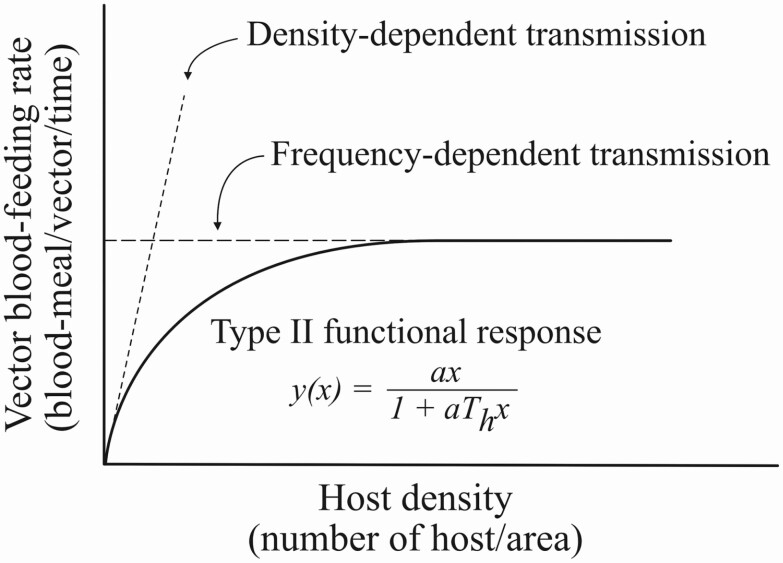
Type II functional response (solid curve) fit by the disc equation (Holling 1959a) is adapted to account for the relationship between the contact rate and host density in vector-borne disease transmission. This approach is a compromise between the density-dependent and frequency-dependent transmission (adapted from Antonovics et al. 1995). Here, the predation rate, *y*(*x*), is the vector’s per capita blood-feeding rate; *a* is the vector’s search rate (also called discovery rate), and × is host density (number of hosts per unit area). The ‘handling time’ per host, *T*_*h*_, includes the average time a mosquito spends to interact with the host, bite the host, and process the bloodmeal after biting (Demers et al. 2018).

Holling (1959a,b, 1965) identified three types of functional responses, although type II is frequently observed in nature (Fig. 4). Type II functional response describes a predation response that rises at a decreasing rate to reach an upper asymptote at a maximum value of prey density (Hassell et al. 1977, Jeschke et al. 2002). A popular expression for this relationship is a disc equation, derived by Holling (1959a; Fig. 4).

Type II functional response has been adopted to describe a relationship between mosquito blood-feeding rate and host density (Antonovics et al. 1995, Miller et al. 2016, Demers et al. 2018). A mosquito vector is comparable to a predator ‘preying’ upon hosts where the predation rate is equivalent to a blood-feeding rate (number of blood meals per vector per unit time). In an area where hosts are sparse (host number in the area is low), the vector needs to spend more time searching for the host, resulting in a lower blood-feeding rate. However, the relationship between blood-feeding rate and host density is nonlinear and follows the disc equation’s curve (Fig. 4) because of the time that the vector must take to accomplish blood-feeding (‘handling time’). A large number of hosts can be quickly located by a vector when there are high host densities, but more time must also be spent ‘handling’ the located hosts (Holling 1959a). As a result, the blood-feeding rate does not increase linearly with the host density but instead reaches an asymptote as the host density increases.

Some predation models also include a predator’s satiation level, determined by factors, such as digestion rate (Rashevsky 1959, Rijn et al. 2005). Satiation can be incorporated as part of the handling time called a ‘digestive pause’ (see review in Jeschke et al. 2002), or as a separate background hunger level that determines the probability of initiating the hunt (Holling 1966, Jeschke et al. 2002). A model proposed by Demers et al. (2018) included mosquitoes’ satiation as a digestive pause within the handling period. Thus, the mosquitoes can only take one bloodmeal per gonotrophic cycle. An alternative model includes a separate background hunger level to determine whether the mosquito will search for multiple hosts within one gonotrophic cycle (multiple blood-feeding behaviors).

Functional response is a mechanistic model (Antonovics et al. 1995) that takes into account the impact of host density and vector’s behaviors on the blood-feeding rate (Miller et al. 2016, Demers et al. 2018). It is an intermediate compromise between the density-dependent and frequency-dependent contact type (Fig. 4) and better explains the real-world transmission process (Antonovics et al. 1995). When the contact rate is in the form of Holling’s type II functional response, the force of infection is also a function of the searching, biting, and digesting/ovipositing efficiency of the vector, as well as the density of the vertebrate host.

#### Vector’s Demand and Host’s Supply for Contacts

When the ratio of vector to host populations, *N*_*v*_*/N*_*h*_, is relatively constant, then all of the contact rate (total contacts in the area of interest per unit time) models can be tuned to give approximately the same results. However, when the vector or host population varies significantly, then the contact rate model must account for the variability in the vector’s demand and host’s supply. The Chitnis dynamic contact rate model balances the actual number of bites based on parameters for vector’s demand and host’s supply for contacts (Chitnis et al. 2008, Manore et al. 2015). The parameter, $\sigma_{vh},$ is the number of bites per unit time a mosquito (species *v*) would ideally want from the host (species *h*). The parameter, $\sigma_{hv},$ is the number of bites that a host (species *h*) can support per unit time for the vectors (species *v*). Thus, the total number of bites all mosquitoes in the population want per unit time or “bite demand” is derived by multiplying $\sigma_{vh}$with the vector population size (*N*_*v*_). The total number of bites all hosts in the population can support per unit time, or ‘bite supply’, is derived by multiplying $\sigma_{hv}$with the vector population size (*N*_*h*_). The total number of host–vector contacts ($C_{vh}$) is half the harmonic mean of the bite supply and demand, or


$$\begin{array}{l} C_{vh}=\frac{\left(\sigma_{vh}N_{v})\;\cdot \;(\sigma_{hv}N_{h}\right)}{\left(\sigma_{vh}N_{v})\;+\;(\sigma_{hv}N_{h}\right)}. \end{array}$$ (4)

**Fig. 5.**
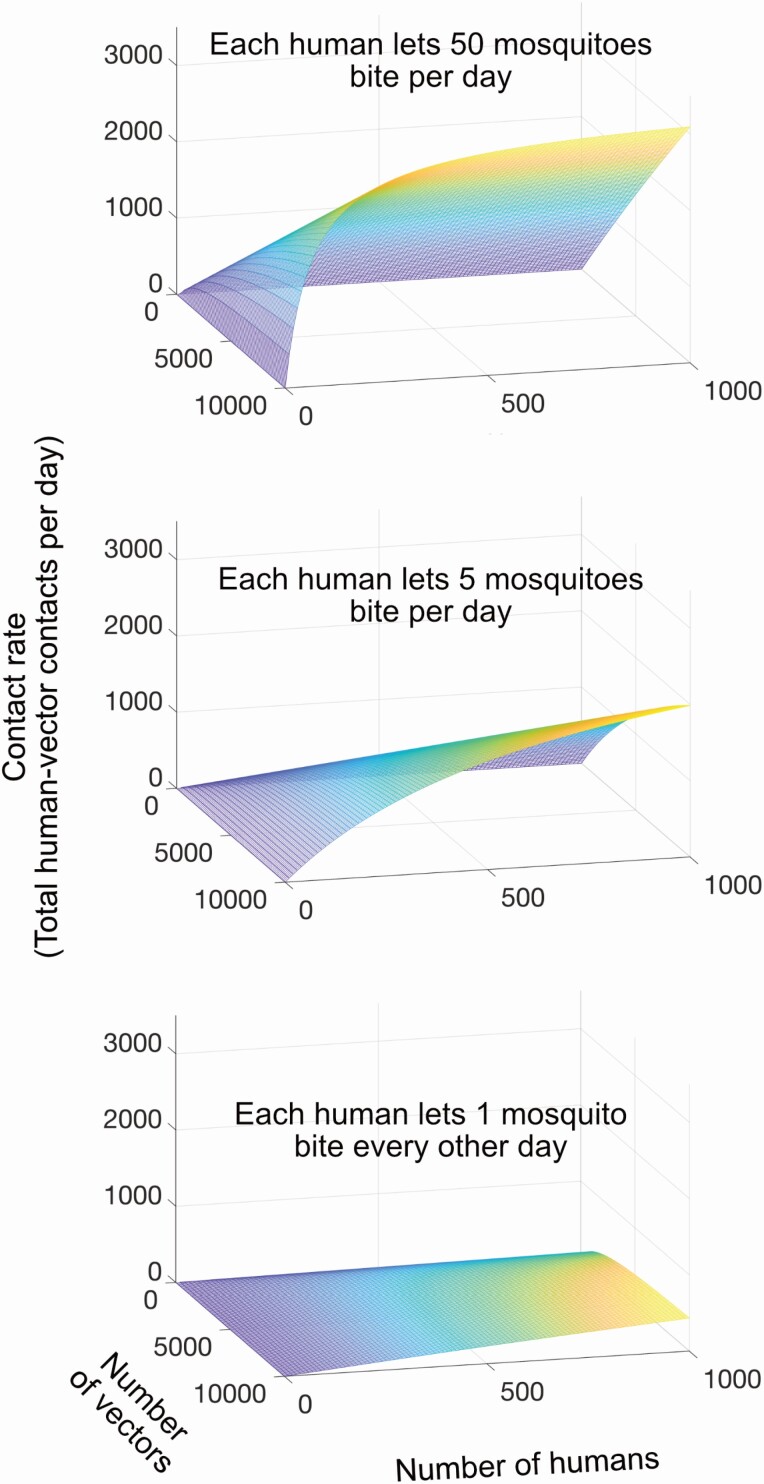
Relationships between contact rate (total contacts per day) and the numbers of vectors and humans when the contact rate is derived are half the harmonic mean of vector’s bite demand and human bite supply (equation 4). In all cases, each mosquito wants to bite a human every three days.

This relationship has the correct limits for the contact rates for both the bite demand $\left(\sigma_{vh}N_{v}\right)$ and supply $\left(\sigma_{hv}N_{h}\right)$ as either population approaches zero or infinity. For example, when the host’s bite supply is zero (either because there are no hosts around, or the hosts do not let mosquitoes bite at all), the resulting host–vector contact rate according to equation (4) is also zero. When the bite supply is large (either because the host population size is large, or each host allows a large number of vector bites per unit time), the resulting contact rate according to equation (4) is also large, approaching the bite supply value as the bite demand increases. Similarly, when the bite demand is high (either because the vector population size is large, or each vector needs a large number of bites per unit time), the resulting contact rate is also high, approaching the bite demand value as the bite supply increases. This relationship also meets the necessary criteria that contact rates are always less than either the bite demand or supply (Fig. 5).

### Feeding Pattern Models

A heterogeneity in pathogen transmission arises primarily from variability in contact rates among hosts and vectors with varying degrees of infectiousness (Kilpatrick et al. 2006). In VBD transmission, contact patterns between vectors and hosts significantly affect disease transmission outcomes (Kilpatrick et al. 2006, Simpson et al. 2011, Harrington et al. 2014, Guelbéogo et al. 2018). Also, contact patterns can be influenced by various biological variables related to both vectors and hosts (‘Factors Influencing Host–Vector Contact’ section). The classic Ross–Macdonald modeling style assumes a simple scenario where a mosquito bites randomly and evenly among vertebrate hosts (Smith et al. 2012). According to a systematic review study (Reiner et al. 2013), 82 and 78% of all mechanistic models simulating mosquito-borne disease transmission published between 1970 and 2010 assumed that blood meals are homogeneously distributed among host sources and that contacts between host and vector are well-mixed, respectively. In the following section, we give examples of how contact patterns are alternatively modeled based on vector feeding behaviors or host behavioral responses.

### Blood Index and Forage Ratio

Biologists determine host–vector contact patterns by analyzing vector blood meals, producing indices such as the Human Blood Index and Forage Ratio. Both indices have been applied in mathematical models to derive contact rate, demonstrating how nonrandom blood-feeding influences disease transmission (Garrett-Jones 1964, Hess et al. 1968).

The Human Blood Index is the *proportion* of mosquito blood meals from humans (Garrett-Jones 1964). To generalize this index to other nonhuman animals, we define blood index, *ø*_*h*_, as the proportion of mosquito blood meals from host species *h*. For a mosquito species *v*, which feeds on multiple host species, we can determine the blood-feeding rate on host species *h* (*b*_*vh*_) by multiplying the mosquito’s total feeding rate *B*_*v*_ (regardless of host species) and the Blood Index for that vertebrate host (*ø*_*h*_), or


$$\begin{array}{l} b_{vh}=\phi_{h}B_{v}. \end{array}$$ (5)

Then, the bite-exposure rate from vector species *v* (the number of bites per host per unit time from vector species *v*) can be derived by multiplying the blood-feeding rate with the number of mosquitoes per host, or


$$\begin{array}{l} e_{vh}=mb_{vh} \end{array}$$ (6)

where *m* is the number of mosquitoes of species *v* per host species *h*.

The Human Blood Index can be influenced by the vector preference to seek out and feed on humans, human availability, and other environmental factors. For example, even though a vector may prefer to feed on humans, the low number of humans in the area could result in a low value of the Human Blood Index. On the other hand, the forage ratio (sometimes called Feeding Preference Index) takes into account the relative number of host species of interest to other host species. It measures the proportion of blood meals belonging to a host species *in relation to* the proportional density of that host in a whole host community (Hess et al. 1968, Hassan et al. 2003, Kilpatrick et al. 2006, Hamer et al. 2011); or


$$\begin{array}{l} \alpha_{h}=\frac{\phi_{h}}{\delta_{h}}, \end{array}$$ (7)

where *α*_*h*_ is the forage ratio on host species *h*, *ø*_*h*_ is the blood index of host species *h*, and $\delta_{h}$ is the density of host species *h* in the area divided by the density of all hosts in the community.

If vectors feed on host species *h* in proportion to their density (opportunistic feeding), the fraction of blood meals from each species will be the same as their relative density, resulting in a forage ratio equal to 1. When the forage ratio is >1, ‘preferred feeding’ or ‘overuse’ of host *h* occurs. When the forage ratio is <1, ‘avoided feeding’ or ‘underuse’ of host *h* takes place (Kilpatrick et al. 2006, Hamer et al. 2009). Note that we prefer the term ‘Forage Ratio over Feeding Preference Index’ to avoid confusion with the ‘Feeding Index’ (as used in Kay et al. 1979, Richards et al. 2006).

The forage ratio was incorporated into multi-host vector-borne pathogen transmission models to investigate the effect that contact patterns have on disease spread (e.g., Simpson et al. 2012, Iacono et al. 2013, Caja Rivera 2019). Results from model analyses revealed that forage ratio in a system where host competence varies across species could cause extreme transmission heterogeneity, substantially impacting pathogen transmission outcomes.

In addition, Yakob (2016) developed a model to describe the nonlinear response of blood index to host relative density and called this relationship ‘behavioral response’, analogous to the functional response. The forage ratio can vary across different host relative densities depending on the shape of the relationship that reflects various vector feeding behaviors. For example, if the vector is anthropophilic, the response curve of the Human Blood Index to human relative density is concave down, analogous to Holling’s class II functional response. Here, the Human Blood Index rises quickly at low relative human density, and eventually, almost all blood meals are from humans even when relative human density is low. For a zoophilic vector, the response curve is concave up. The Human Blood Index rises slowly at low relative human density, and only when humans are the sole host does the Human Blood Index approach one. This type of model provides a general framework to incorporate various host–vector contact patterns that could vary due to their relative density and behaviors, and explore its impact on infectious disease epidemiology.

#### Modeling Host Defensiveness and Ideal Free Distribution

The forage ratio is sometimes called a feeding preference index, a misleading term as it implies that the vector is the only actor in a two-way relationship. In theory, a shift in the forage ratio can result from either vector or host behaviors. When the forage ratio is greater than one, it indicates that either the vector prefers to bite the host or that the host exhibits low protective or defensive behaviors (Edman and Scott 1987, Darbro and Harrington 2007). Even though the vector shows an innate preference for certain host species or groups (see ‘Mosquito Innate Host Preference’ section), other influencing variables can be equally important in driving heterogeneity of the forage ratio and blood index.

The host protective and defensive behaviors against vector bites are not often included in disease transmission models. Laboratory studies have shown that increasing vector density cause increases in host defensiveness, which reduces successful feeding (Kale et al. 1972, Webber and Edman 1972, Waage and Nondo 1982). This phenomenon is called host-mediated density-dependent interference (Kelly et al. 1996). Kelly proposed a model adapted from a predator–prey system (Sutherland 1983) to explain the relationship between vector feeding success, density-dependent interference, and host permissiveness. Specifically, the vector’s feeding success increases with host intrinsic permissiveness when there is only one biting individual vector (Kelly and Thompson 2000). However, when there is more than one vector feeding on the host (vector to host ratio, or biting density > 1), the vector’s feeding success starts to drop. The rate of this decrease depends on the strength of the interference among vectors (such as through host-defensive behaviors). The higher the interference, the faster feeding success drops as more and more vectors compete to feed on the host. This level of interference was proposed to have a power relationship with the ratio of biting vectors on individual hosts. This hypothesis can be expressed by the equation


$$\begin{array}{l} G_{i}=\frac{Q_{i}}{N_{i}^{m\;}}, \end{array}$$ (8)

where *G*_*i*_ is the per capita feeding success of vectors on a host *i*, *Q*_*i*_ is the host’s intrinsic permissiveness, *N*_*i*_ is the vector biting density on host *i*, and *m* is an interference constant which determines the level of interference among competing vectors (Kelly et al. 1996).

Kelly suggested that density-dependent interference would be minimized if vectors obey the Ideal Free Distribution (IFD; Kelly et al. 1996). IFD is a theory that was developed to explain the distribution of animals around their environment or patches of resources (Fretwell and Lucas 1969). IFD assumes that the organisms are ‘ideal’ in their ability to assess each resource patch’s quality, and ‘free’ to move between patches without restriction or cost (Sutherland 1983). The original IFD predicts that animals will distribute themselves among resource patches to balance resource abundance and interference level in each patch so that each animal will experience equal gain or feeding success (Sutherland 1983). Since the inception of the theory, multiple modifications to the IFD models were proposed (see review: Křivan et al. 2008).

IFD has been applied to explain how blood-feeding insects distribute themselves among vertebrate hosts (Kelly et al. 1996, Kelly and Thompson 2000). If a vector does follow the IFD, then ideally, they would distribute their numbers among available hosts so that they experience equal feeding success for all hosts (i.e., *G*_*i*_ is the same for all hosts *i*). In this way, one can derive the vector’s blood index of each host species based on host permissiveness, vector to host ratio, and vector interference level. Including IFD theory in disease transmission models add realism to models that seek to predict the effect of these ecological parameters on disease spread (Kelly and Thompson 2000, Lord 2004). Unfortunately, whether the IFD model can be used to accurately predict the observed vector’s feeding patterns in nature is unknown. More field data are needed in order to determine whether IFD is an appropriate model for blood-feeding insects and their host resources.

## Discussion and Summary

A critical goal of monitoring vector-borne diseases is quantifying transmission risk to humans in the presence of vectors. A comprehensive framework for assessing transmission risk is important for identifying the relative importance of various extrinsic and intrinsic drivers of risk, and designing and implementing strategic control efforts to mitigate these risks, especially in limited-resource settings.

Vector density is an obvious contributor to mosquito-borne disease risk that is straight-forward to measure. All things being equal, the more mosquitoes there are in an area, the higher the risk of the infection in many situations (Moreno et al. 2007, Galardo et al. 2009, Bowman et al. 2014). However, as we have shown, vector density is not always directly proportional to disease risk. Contact between mosquitoes and humans, via mosquito bites and blood-feeding, directly drives mosquito-borne disease transmission (Fig. 4). Assessing disease risk solely as a function of vector density also ignores the human side of the transmission interaction. We conclude that a more useful depiction of disease transmission dynamics requires the inclusion of contact rates to capture both mosquito and human roles in the equation.

Unfortunately, human–mosquito contact is not often considered in disease risk assessment because we lack practical field methods to measure it (Lima et al. 2014). Only a few approaches have been used to characterize human–mosquito contact. Human landing catch (HLC), involving human volunteers collecting mosquitoes that land on them to feed, is the traditional gold standard method to monitor mosquito bite-exposure levels in malaria transmission (Wong et al. 2013, Kenea et al. 2016). Although a well-designed HLC study could be used to approximate contact rate with night biters such as *Anopheles* spp. (Moiroux et al. 2014, Martin et al. 2020, Monroe et al. 2020), other vectors such as *Aedes* spp. bite during the day when humans may actively interrupt or avoid bites. In addition to being unethical, since they expose people to potentially infected vectors (Ndebele and Musesengwa 2012), HLC estimates are likely to be biased, especially in the case of *Aedes*–human contact rates, by variation in housing infrastructure, human behaviors, and lifestyle differences that cannot be captured easily by an HLC experiment (Reiter et al. 2003, Haenchen et al. 2016, Ndenga et al. 2017).

Attempts to estimate components of contact rate, such as vector blood-feeding rate, includes Scott et al.’s (1993, 2000) histological technique to estimate the number of blood meals and time since blood-feeding based on the level of blood digestion and ovarian development in *Ae. aegypti*, compared to known laboratory standards. This technique can give a fair estimate of the blood-feeding rate but is time-consuming, requiring preparations of many mosquito samples for histological analysis. Other studies have used PCR-based DNA profiling to construct allelic profiles of research participants and compare them with the profiles of blood found in mosquitoes (De Benedictis et al. 2003, Harrington et al. 2014, Liebman et al. 2014). Results from these studies confirm the mosquito’s multiple-feeding behavior but do not provide an estimate of blood-feeding rates. Finally, some studies used the serum concentration of anti-salivary gland extract (SGE) antibodies as a biomarker of bite-exposure (Fontaine et al. 2011, Londono-Renteria et al. 2013). Similarly, this method provides only the level of bite exposure, not rate.

Survey tools have been used to characterize and estimate mosquito bite-exposure levels in humans. Surveys often entail asking participants to describe their bite-exposure levels in qualitative terms such as ‘every day’ or ‘rarely’ (Logan et al. 2010, Dowling et al. 2013, Halasa et al. 2014). One study asked respondents to quantitatively indicate the number of mosquito bites they received within the past 24 h, resulting in estimates for bite-exposure rate (Thongsripong et al. 2020). However, self-reported mosquito bite-exposure rate estimates possess numerous biases (Thongsripong et al. 2020) and require further studies to evaluate specificity and sensitivity compared to other field methods.

Better field methods to assess human-mosquito contact rates are critical to determine the appropriate disease transmission model for a particular biological question. Ideally, these methods need to consider the heterogeneity in both human and mosquito factors that influence the contact rates and generate estimates useful for mathematical models of disease transmission. Mathematical models can provide valuable insights into a complex biological system, such as VBD transmission, and practically inform disease prevention and control. For the model predictions to be valid, its structure needs to include the appropriate parameters, and field observations and experimental studies must inform their values. In addition, a model should only be as complex as needed and constructed based on the question of interest because unnecessary complexity can obscure fundamental structure and transparency. The gap in our knowledge of human–mosquito contact rates is an opportunity to integrate empirical, experimental, and theoretical approaches for a suitable model formulation and strategic data collection. To that end, our model formulation points to several avenues of future studies that would be strategic (Box 1).

Box 1 **: Strategic research areas identified by human–vector contact modeling framework**

These models reframe disease transmission risk dynamics in terms of key contact rates (bite-exposure/blood-feeding) instead of other traditional measures, such as mosquito density. This framework highlights opportunities for several follow-up areas of study that would be particularly strategic:

1. Quantifying contact rates in the field in response to extrinsic and intrinsic drivers. A standardization and/or the development of new field methods to quantify both mosquito biting rate as well as host bite exposure rate is an important next step to better disentangle drivers of contact rates and transmission dynamics, such as urbanization, temperature, and human behavior.
2. Developing mathematical models that explicitly address critical biological questions. We identified several potentially important factors that could be expanded on explicitly from our initial modeling framework, for example, explicit exploration of the importance of multiple blood feeding behaviors for a given vector, and the implementation of models for host protective measures.
3. Empirical insights into human–mosquito contact rates. Laboratory experiments, semifield and field observations, and other studies are critical to help resolve factors influencing contact rates and disease transmission, for example, the potential importance of multiple blood feedings, the impacts of infection status of either the human host or the vector on biting behaviors, how human behavior impacts contact rates, and how temperature and other environmental changes impact mosquito biting rate.

Ideally empirical and modeling studies will be integrated by transdisciplinary teams of experts that can specify, parameterize and test new models to distinguish between alternative formulations and determine which factors most influence host-vector contact rates following the suggestions for data, models and experiments (1–3 above) for specific host-vector-pathogen systems.

Field investigations yield two distinct estimates of contact rate: a vector’s per capita blood-feeding rate or a host’s per capita bite-exposure rate (with or without blood-feeding). As discussed in ‘What is a Human–Mosquito Contact?’ section, these rates are mathematically distinct because not all mosquito bites result in blood-feeding. They also differ biologically because each represents an alternate direction for disease transmission. The transmission from host to vector is a function of the blood-feeding rate, whereas the transmission from vector to host is a function of the bite-exposure rate. Currently, a majority of models use only the blood-feeding rate (as a reciprocal of the vector’s gonotrophic cycle length) to calculate the rate of disease spread, while the bite-exposure rate is considered as a derivation of the vector’s blood-feeding frequency. Although this assumption seems acceptable given our limited knowledge about the two rates, the distinction could be important for multiple reasons.

First, variation in human behavior, movement, culture, lifestyle, and socioeconomic background substantially influence the levels of mosquito bite exposure. Given a similar level of mosquito density, the individual or population differences in these characteristics determine the risk of becoming infected with mosquito-borne diseases. By characterizing the bite-exposure rate instead of the blood-feeding rate, we can directly investigate how changes in human behavior and society influence mosquito-borne disease transmission. This information is indispensable if we want to predict how the changing environment, such as urbanization, poverty, and climate change, impacts human behaviors that affect mosquito-borne disease transmission. Our approach provides a framework for strategic field and laboratory studies to elucidate the roles of the environment and host behavior in bite-exposure rate, and how to more explicitly model these potential drivers of disease dynamics (Box 1)

Second, not all mosquito bites result in successful blood-feeding. For example, a mosquito might attempt to bite (probe) multiple times before imbibing blood. As a result, the mosquitoes’ total blood-feeding and humans’ total bite-exposure could be very different. Thus, calculating bite-exposure rates from the frequency of blood-feeding is not appropriate, especially for skittish day biters such as *Ae. aegypti* (Scott and Takken 2012). Field data to determine the significance of the difference between these two rates are needed, as are complementary laboratory and field experiments to better characterize the importance of mosquito feeding behaviors, including the influence of infection status (of host or vector), on contact rates and pathogen transmission (Box 1).

This review article summarizes and synthesizes current knowledge of host–vector contact, with an emphasis on mosquito bite dynamics. We provide consistent biological and mathematical definitions of host–mosquito contact rate, blood-feeding rate, and bite-exposure rates. We explain why it is essential to differentiate these terms and present how various biological and environmental factors can influence them. We described how host–mosquito contact parameters are typically incorporated into classic VBD compartmental models. We then connect these parameters to ecological concepts, such as functional response, blood index, and forage ratio to model host–vector contact rates.

Importantly, we illustrate that host–vector contact is a critical meeting point where multiple influencing factors interact to drive disease transmission. We clarify the mechanisms driving the spread of infections by reframing the transmission process around this parameter. Vectors and vertebrate hosts continuously react to changes in one another and in the environment, resulting in critical variations in contact rate underlying disease transmission risk. This challenges the common modeling assumption that mosquitoes bite at a fixed rate determined by the duration of their gonotrophic cycle. Our framework identifies critical gaps in current knowledge of human–mosquito contact. It emphasizes the need to develop field-based approaches to quantify and characterize the contact rate in mathematical models in order to test which factors impact contact rate to more accurately predict VBD risk. This framing should help VBD professionals think about how well interventions focused on population size reduction actually decrease host-vector contact. Overall, this synthesis provides a logical framework to understand the mechanisms driving VBD emergence to help guide future research directions and better inform disease prevention and control.
